# Supplementary material for: Piezoceramic membrane with built-in ultrasound for reactive oxygen species generation and synergistic vibration anti-fouling
Source: Nat Commun. 2024 Jun 6;15:4845. doi: 10.1038/s41467-024-49266-1 (PMC11156986; doi:10.1038/s41467-024-49266-1)
Supplement: Supplementary file 1 — Supplementary Information [file 41467_2024_49266_MOESM1_ESM.pdf]

Supplementary Information

Piezoceramic membrane with built-in ultrasound for reactive oxygen species generation and synergistic vibration anti-fouling

Yang Zhao[1,2*], Feng Yang[1,2], Han Jiang[1,2], Guandao Gao[3,4]

[1]*School of Energy and Environment, Southeast University, Nanjing, 210096, China*

[2]*State Key Laboratory of Environmental Medicine Engineering, Ministry of Education, Southeast University, Nanjing, 210096, China*

[3]*State Key Laboratory of Pollution Control and Resource Reuse, School of The Environment, Nanjing University, Nanjing, 210023, China*

[4]*Research Center for Environmental Nanotechnology (ReCENT), Nanjing University, Nanjing, 210023, China*

* Corresponding author, Yang Zhao, E-mail: zhaoyangseu@seu.edu.cn

Contents

- Number of Pages: 12
- Pages 3-5: Supplementary Fig. 1-3
- Pages 6: Supplementary Table 1, Supplementary Fig. 4
- Pages 7-11: Supplementary Fig. 5-11
- Pages 12: Supplementary References

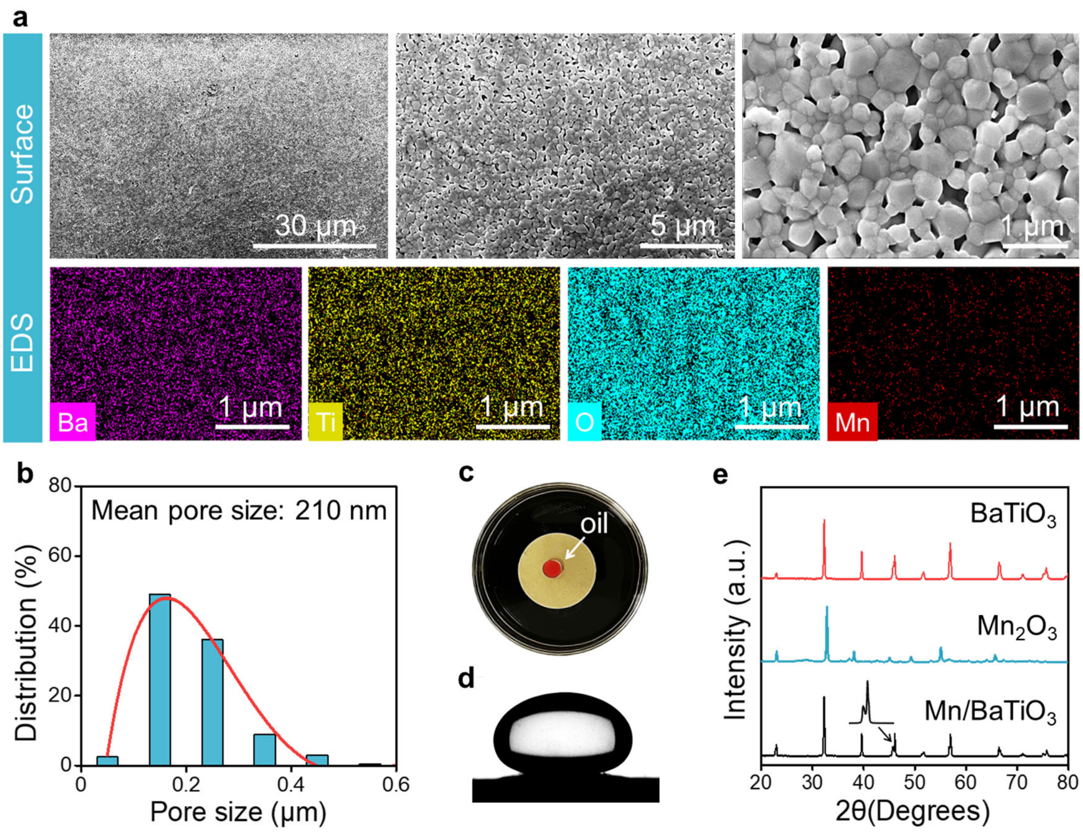

Supplementary Fig. 1. Mn/BaTiO$_3$ Piezoelectric Membrane Characterization. a ESEM and corresponding EDS elemental mapping of Mn/BaTiO$_3$ piezoelectric membrane (corresponding ESEM images scale bar, 1 μm). **b** Pore size distribution. **c-d** Underwater oil contact angle of the membrane. **e** XRD patterns of the samples.

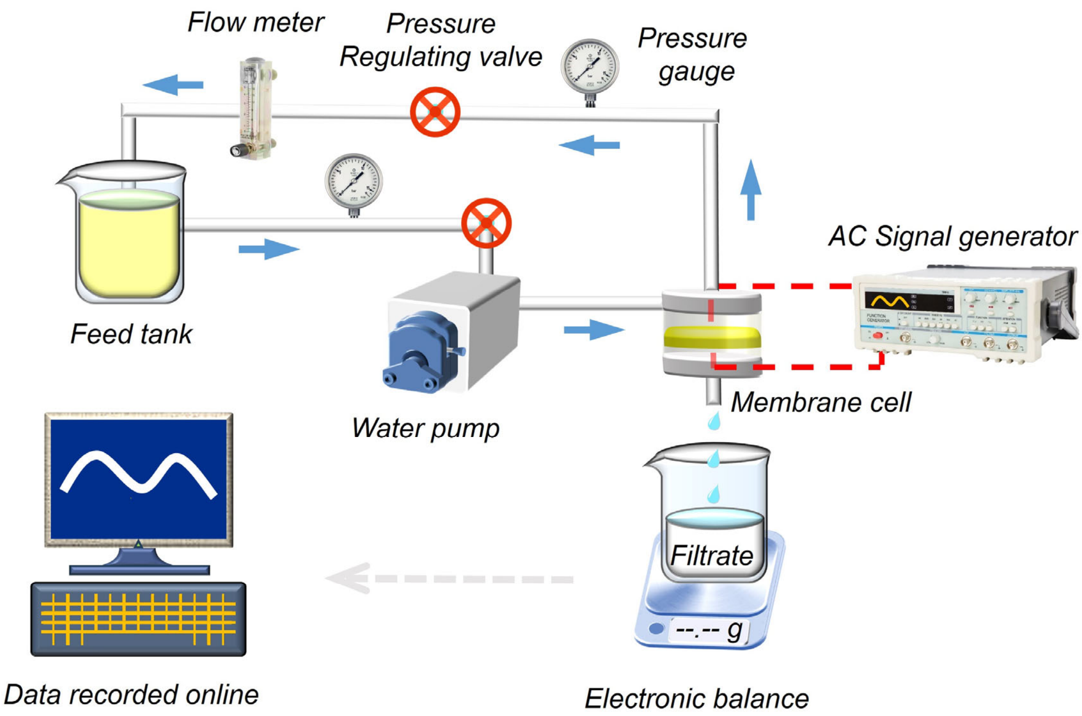

Supplementary Fig. 2. Lab-scale cross-flow filtration and real-time recording system schematic.

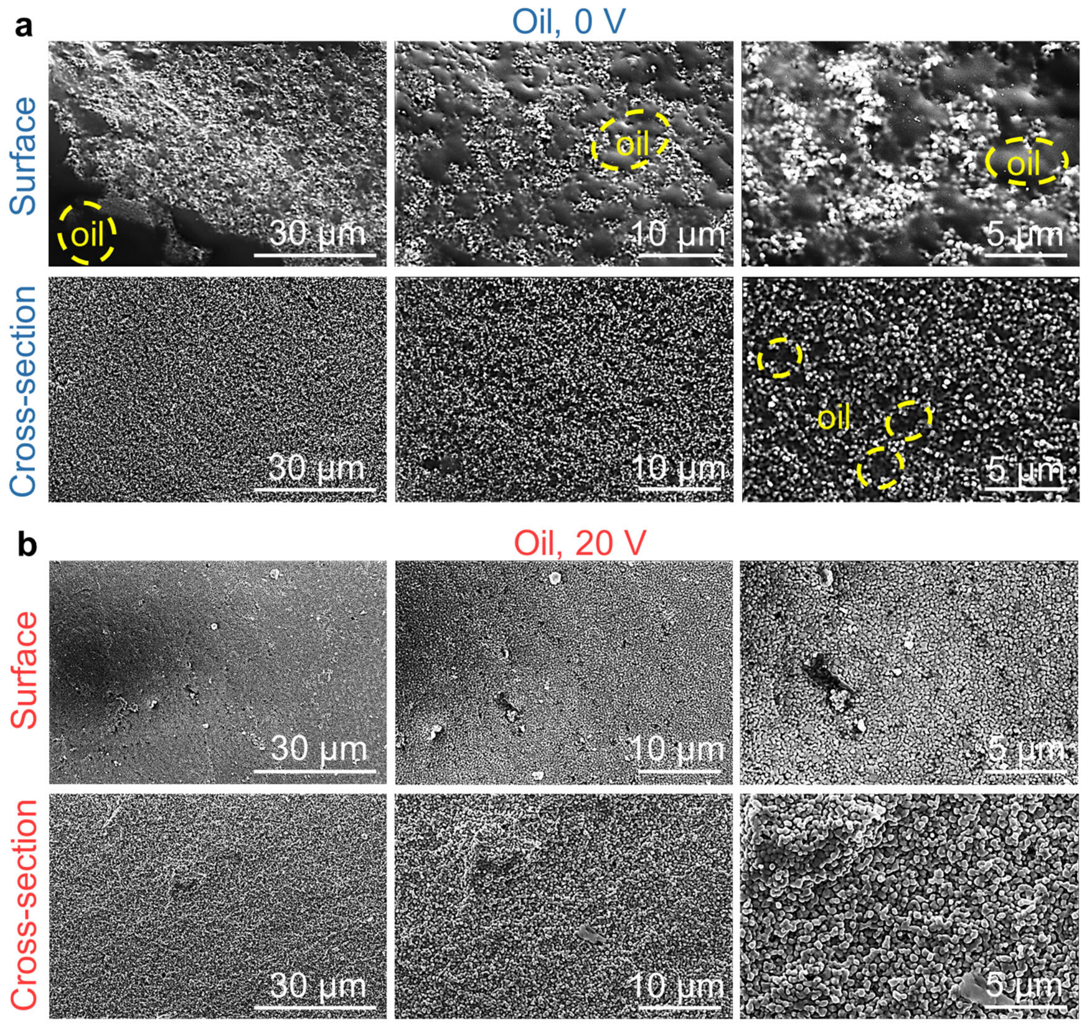

Supplementary Fig. 3. ESEM images of Mn/$BaTiO_3$ piezoelectric membranes fouled by oil under different AC conditions (265 kHz, **a** 0 V, **b** 20 V). The representative foulants are delineated with yellow dashed lines for clarity.

Supplementary Table 1. The anti-fouling and oil separation performance of the reported membranes and the proposed Mn/BaTiO3 piezoelectric membrane.

Membrane	Flux (L $m^{-2}h^{-1}$)	Oil rejection (%)	Anti-fouling efficiency (%)*	Single running time (min)	Reference
PDA/RGO/HNTs	~70	~99	~75	5	1
HKUST-1(MOFs)/PDA@SM	140-200	~99	~50	20	2
SiC-Al_2O_3	167-360	-	~60	~20	3
F_{10}-hGO	310	>98	70.5	30	4
CNTs-PAN	~160	95	80	60	5
FePc/PVDF	158.94	~97	~90	120	6
ZnO_2	~130	>99.7	~60	180	7
Mn/$BaTiO_3$	~91	98.4	91	180	This work

* Only single-run anti-fouling test results are considered, excluding scenarios where membranes are cleaned and retested after fouling.

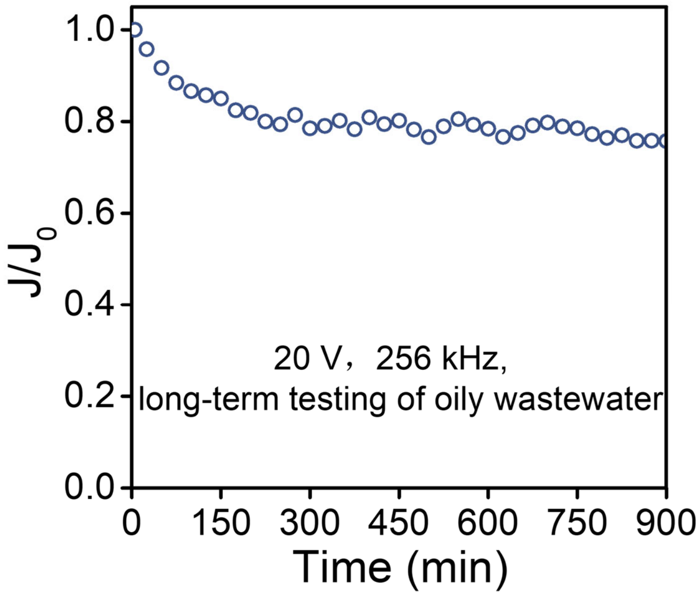

Supplementary Fig. 4. Long-term anti-fouling test of Mn/BaTiO3 piezoelectric membrane for oil wastewater (2500 ppm) treatment.

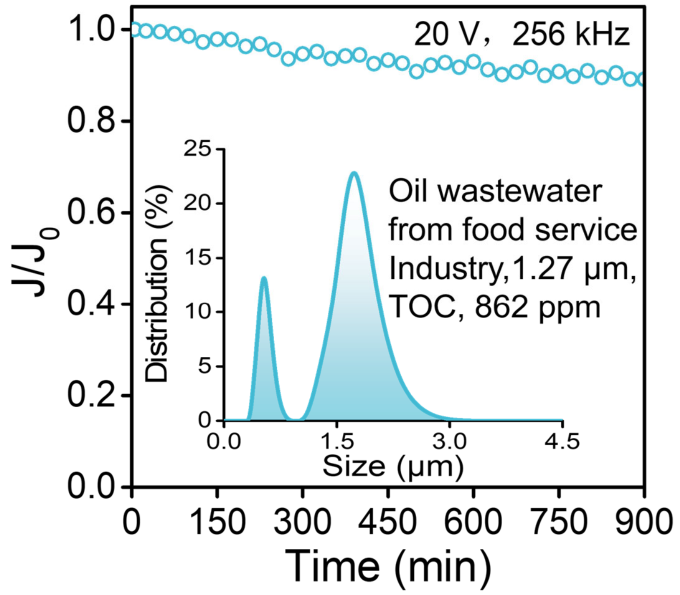

Supplementary Fig. 5. Long-term anti-fouling test of the Mn/$BaTiO_3$ piezoelectric membrane for real industrial oil wastewater treatment (oil particle size distribution, insert).

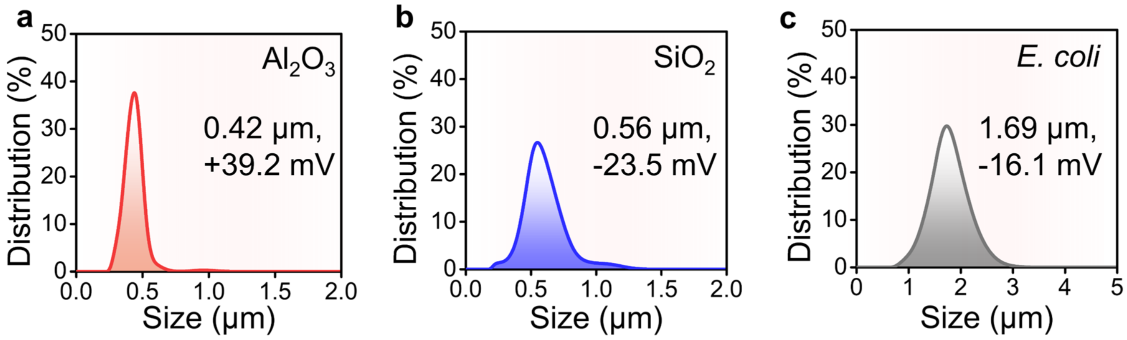

Supplementary Fig. 6. Particle size distribution and zeta potential of the other typical foulants (**a** Al_2O_3, **b** SiO_2, **c** *E. coli*).

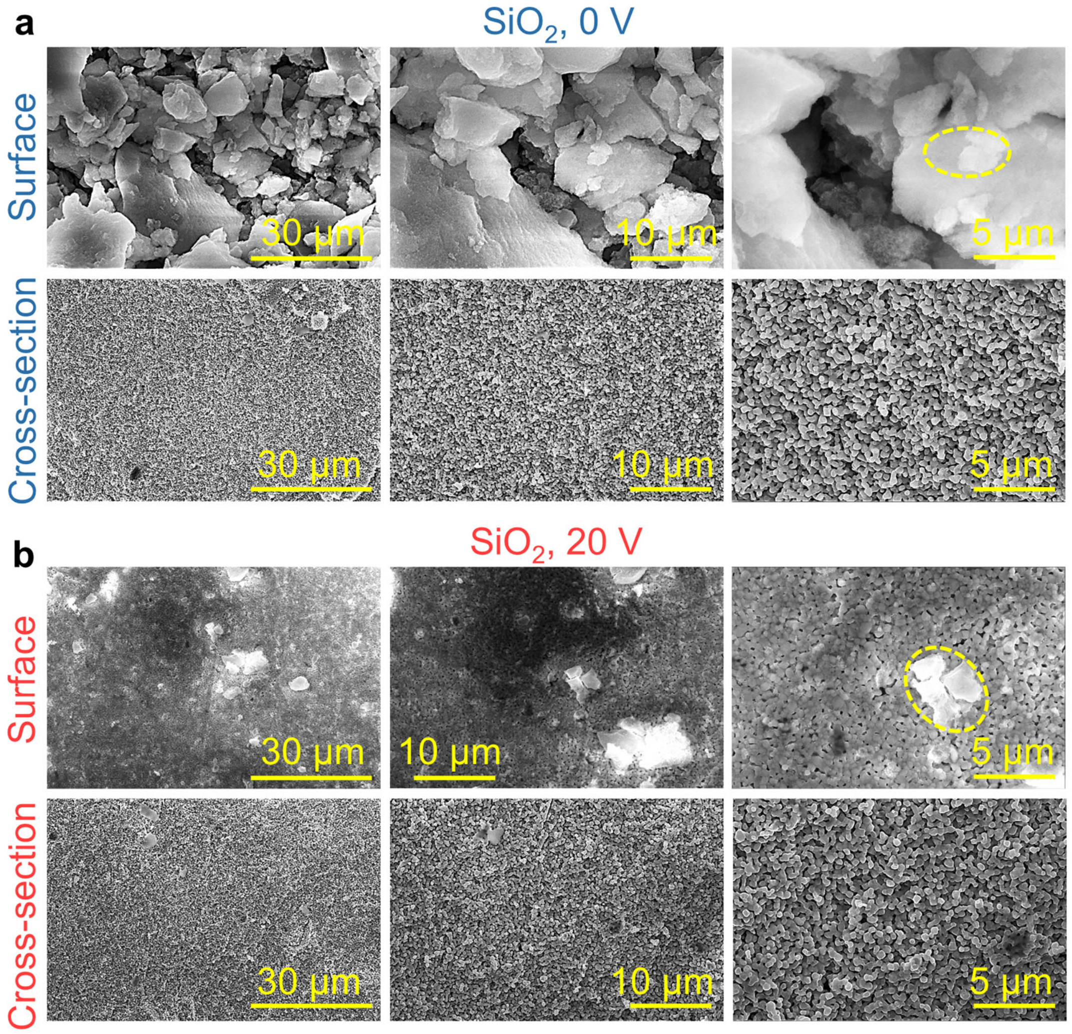

Supplementary Fig. 7. ESEM images of the Mn/$BaTiO_3$ piezoelectric membranes fouled by SiO_2 (-) under different voltages (265 kHz, **a** 0 V, **b** 20 V). The representative foulants are delineated with yellow dashed lines for clarity.

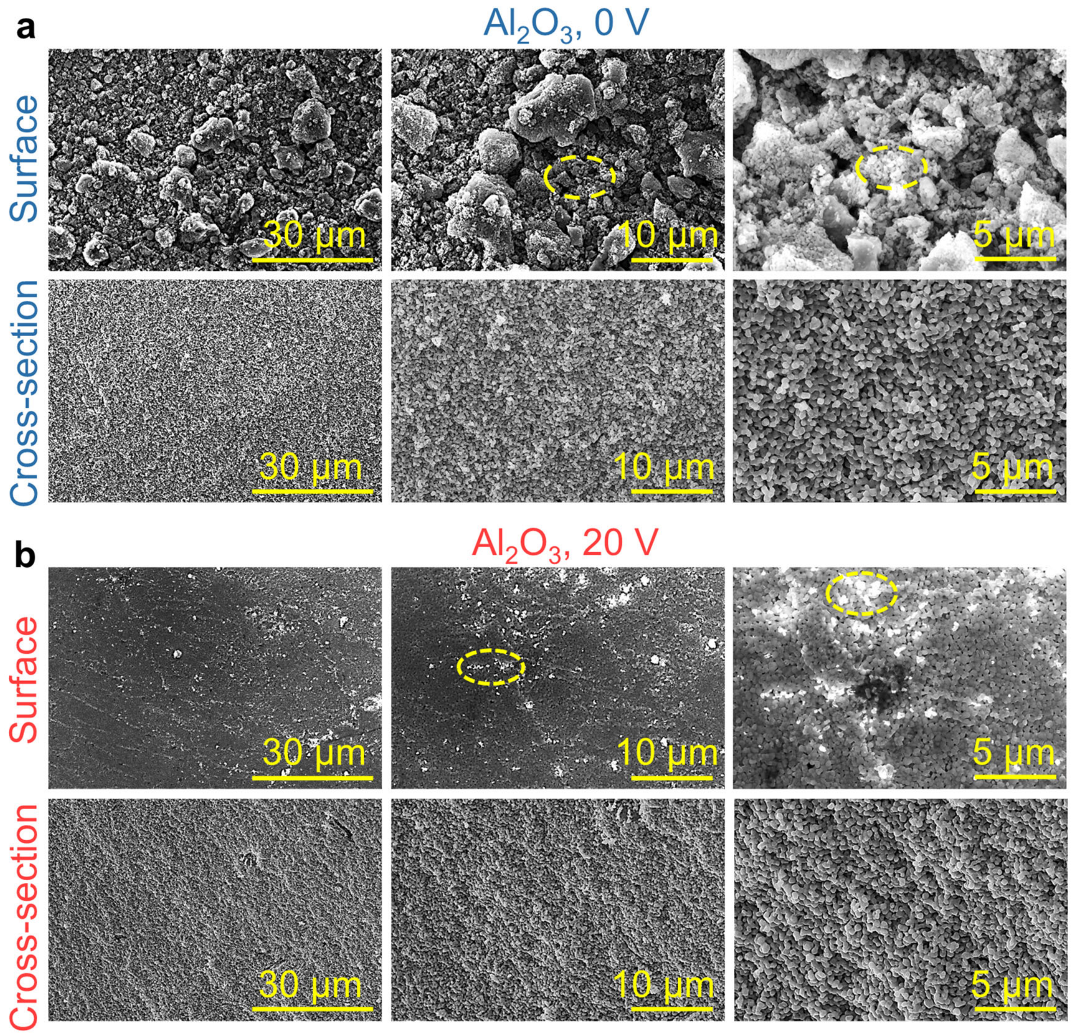

Supplementary Fig. 8. ESEM images of Mn/$BaTiO_3$ piezoelectric membranes fouled by Al_2O_3 (+) under different voltages (265 kHz, **a** 0 V, **b** 20 V). The representative foulants are delineated with yellow dashed lines for clarity.

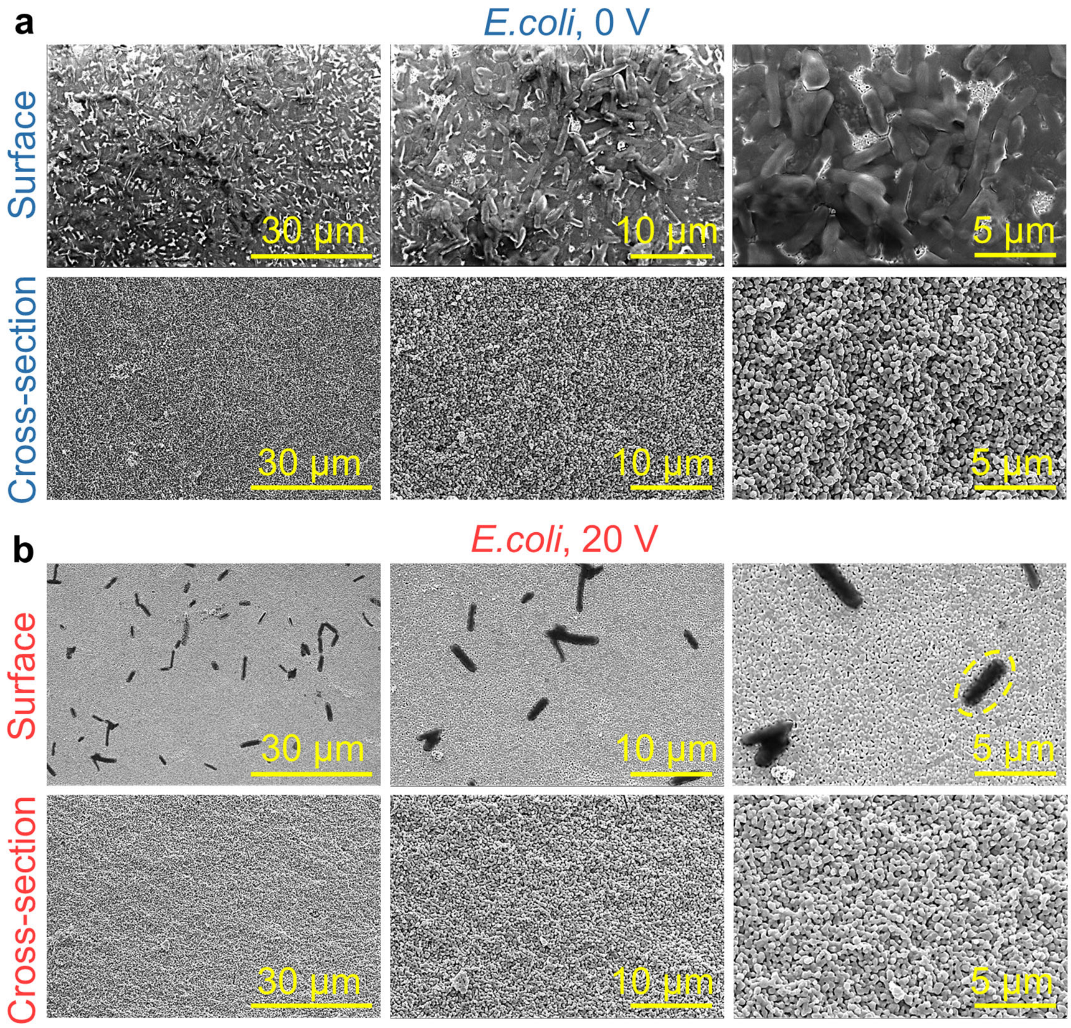

Supplementary Fig. 9. ESEM images of Mn/$BaTiO_3$ piezoelectric membranes fouled by *E. coli* under different voltages (265 kHz, **a** 0 V, **b** 20 V). The representative foulants are delineated with yellow dashed lines for clarity.

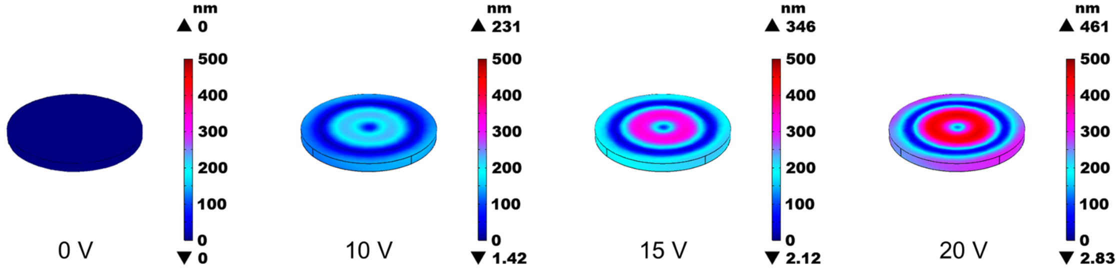

Supplementary Fig. 10. Finite element simulation results showing total displacement in Mn/$BaTiO_3$ piezoelectric membranes stimulated by various AC voltages at 265 kHz.

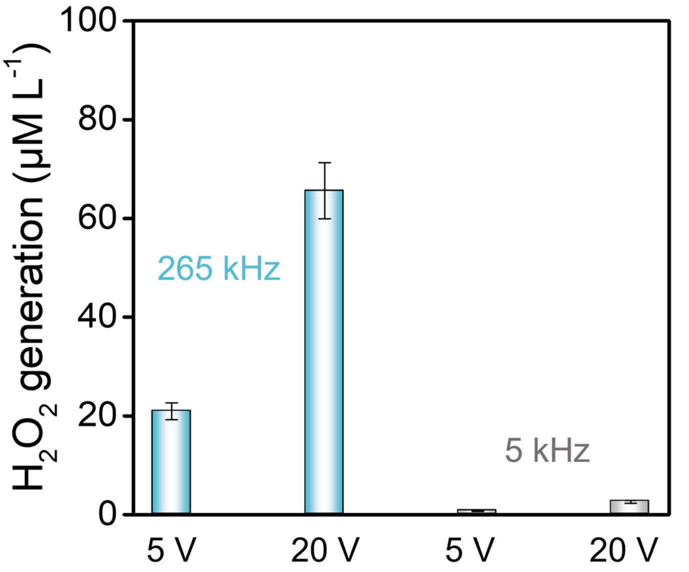

Supplementary Fig. 11. H_2O_2 generated by the ultrasonic vibrations of the Mn/$BaTiO_3$ piezoelectric membranes under different AC conditions. Error bars represent the standard deviation of three measurements.

As illustrated in Supplementary Fig. 11, the membrane built-in ultrasonic vibration in water generated H_2O_2, which is correlated with the applied AC frequency and voltage. The maximum production of H_2O_2 was achieved within 5 min at the optimal membrane resonance frequency of 265 kHz, reaching 65.6±5.7 μM L^{-1}. This result is consistent with the membrane anti-fouling performance. Based on the different ROS detected during the piezoelectric ultrasonic vibration membrane process, ROS interrelations could be proposed as:

$$2H_2O \rightarrow 2\cdot OH + H_2$$

$$2\cdot OH \rightarrow H_2O_2$$

$$H_2O_2 + \cdot OH \rightarrow {}^1O_2 + H_2O$$

Supplementary References

1. Liu YC*, et al.* A mussel-induced method to fabricate reduced graphene oxide/halloysite nanotubes membranes for multifunctional applications in water purification and oil/water separation. *Chemical Engineering Journal* **336**, 263-277 (2018).
2. Deng YY, Bian HZ, Dai M, Liu X, Peng CS. Underwater superoleophobic HKUST-1/PDA@SM membrane with excellent stability and anti-fouling performance for oil-in-water emulsion separation. *Journal of Membrane Science* **678**, 121655 (2023).
3. Chen M, Heijman SGJ, Luiten-Olieman MWJ, Rietveld LC. Oil-in-water emulsion separation: Fouling of alumina membranes with and without a silicon carbide deposition in constant flux filtration mode. *Water Research* **216**, 118267 (2022).
4. Yang C*, et al.* Antifouling graphene oxide membranes for oil-water separation via hydrophobic chain engineering. *Nature Communications* **13**, 7334 (2022).
5. Tian M, Liao Y, Wang R. Engineering a superwetting thin film nanofibrous composite membrane with excellent antifouling and self-cleaning properties to separate surfactant-stabilized oil-in-water emulsions. *Journal of Membrane Science* **596**, 117721 (2020).
6. Chen FT, Shi XX, Chen XB, Chen WX. An iron (II) phthalocyanine/poly(vinylidene fluoride) composite membrane with antifouling property and catalytic self-cleaning function for high-efficiency oil/water separation. *Journal of Membrane Science* **552**, 295-304 (2018).
7. Wang X, Sun K, Zhang G, Yang F, Lin S, Dong Y. Robust zirconia ceramic membrane with exceptional performance for purifying nano-emulsion oily wastewater. *Water Research* **208**, 117859 (2022).
