# Supplementary material for: Piezoceramic membrane with built-in ultrasound for reactive oxygen species generation and synergistic vibration anti-fouling
Source: Nat Commun. 2024 Jun 6;15:4845. doi: 10.1038/s41467-024-49266-1 (PMC11156986; doi:10.1038/s41467-024-49266-1)
Supplement: Supplementary file 3 — Reporting Summary [file 41467_2024_49266_MOESM3_ESM.pdf]

nature portfolio

Corresponding author(s): Yang Zhao

Last updated by author(s): May 24, 2024

Reporting Summary

Nature Portfolio wishes to improve the reproducibility of the work that we publish. This form provides structure for consistency and transparency in reporting. For further information on Nature Portfolio policies, see our Editorial Policies and the Editorial Policy Checklist.

Statistics

For all statistical analyses, confirm that the following items are present in the figure legend, table legend, main text, or Methods section.

n/a	Confirmed	
☐	☒	The exact sample size (n) for each experimental group/condition, given as a discrete number and unit of measurement
☐	☒	A statement on whether measurements were taken from distinct samples or whether the same sample was measured repeatedly
☐	☒	The statistical test(s) used AND whether they are one- or two-sided<br>*Only common tests should be described solely by name; describe more complex techniques in the Methods section.*
☐	☒	A description of all covariates tested
☐	☒	A description of any assumptions or corrections, such as tests of normality and adjustment for multiple comparisons
☐	☒	A full description of the statistical parameters including central tendency (e.g. means) or other basic estimates (e.g. regression coefficient) AND variation (e.g. standard deviation) or associated estimates of uncertainty (e.g. confidence intervals)
☐	☒	For null hypothesis testing, the test statistic (e.g. F, t, r) with confidence intervals, effect sizes, degrees of freedom and P value noted<br>*Give P values as exact values whenever suitable.*
☐	☒	For Bayesian analysis, information on the choice of priors and Markov chain Monte Carlo settings
☐	☒	For hierarchical and complex designs, identification of the appropriate level for tests and full reporting of outcomes
☐	☒	Estimates of effect sizes (e.g. Cohen's d, Pearson's r), indicating how they were calculated

Our web collection on statistics for biologists contains articles on many of the points above.

Software and code

Policy information about availability of computer code

Data collection	No computer code is involved
Data analysis	No computer code is involved

For manuscripts utilizing custom algorithms or software that are central to the research but not yet described in published literature, software must be made available to editors and reviewers. We strongly encourage code deposition in a community repository (e.g. GitHub). See the Nature Portfolio guidelines for submitting code & software for further information.

Data

Policy information about availability of data

All manuscripts must include a data availability statement. This statement should provide the following information, where applicable:

- Accession codes, unique identifiers, or web links for publicly available datasets
- A description of any restrictions on data availability
- For clinical datasets or third party data, please ensure that the statement adheres to our policy

The data supporting the findings of the study are included in the main text and supplementary information files. Raw data can be obtained from the corresponding author upon request.

Research involving human participants, their data, or biological material

Policy information about studies with human participants or human data. See also policy information about sex, gender (identity/presentation), and sexual orientation and race, ethnicity and racism.

Reporting on sex and gender	We don't have related issues.
Reporting on race, ethnicity, or other socially relevant groupings	We don't have related issues.
Population characteristics	We don't have related issues.
Recruitment	We don't have related issues.
Ethics oversight	We don't have related issues.

Note that full information on the approval of the study protocol must also be provided in the manuscript.

Field-specific reporting

Please select the one below that is the best fit for your research. If you are not sure, read the appropriate sections before making your selection.

☐ Life sciences ☐ Behavioural & social sciences ☒ Ecological, evolutionary & environmental sciences

For a reference copy of the document with all sections, see nature.com/documents/nr-reporting-summary-flat.pdf

Ecological, evolutionary & environmental sciences study design

All studies must disclose on these points even when the disclosure is negative.

Study description	This study reports a Mn/BaTiO3 piezoceramic membrane with effective anti-fouling performance. It exhibits universal anti-fouling capabilities, effectively reducing high-concentration oil, bacteria, and charged inorganic colloidal particle pollutants during water treatment. Through built-in ultrasonic vibration, it generates reactive oxygen species, providing new insights into the anti-fouling mechanisms for organic and inorganic wastewater and advancing the development of piezoelectric catalytic membranes.
Research sample	We don't have related issues.
Sampling strategy	We don't have related issues.
Data collection	The relevant sample test data is automatically recorded and collected by the computer.
Timing and spatial scale	We don't have related issues.
Data exclusions	We don't have related issues.
Reproducibility	All experimental tests are repeatable.
Randomization	We don't have related issues.
Blinding	We don't have related issues.

Did the study involve field work? ☐ Yes ☒ No

Reporting for specific materials, systems and methods

We require information from authors about some types of materials, experimental systems and methods used in many studies. Here, indicate whether each material, system or method listed is relevant to your study. If you are not sure if a list item applies to your research, read the appropriate section before selecting a response.

Materials & experimental systems

n/a	Involved in the study
☒	☐ Antibodies
☒	☐ Eukaryotic cell lines
☒	☐ Palaeontology and archaeology
☒	☐ Animals and other organisms
☒	☐ Clinical data
☒	☐ Dual use research of concern
☒	☐ Plants

Methods

n/a	Involved in the study
☒	☐ ChIP-seq
☒	☐ Flow cytometry
☒	☐ MRI-based neuroimaging

Plants

Seed stocks	We don't have related issues.
Novel plant genotypes	We don't have related issues.
Authentication	We don't have related issues.
